# Overviews on the Progress of Flowable Dental Polymeric Composites: Their Composition, Polymerization Process, Flowability and Radiopacity Aspects

**DOI:** 10.3390/polym14194182

**Published:** 2022-10-05

**Authors:** Evangelia C. Vouvoudi

**Affiliations:** Laboratory of Polymers and Dyes Chemistry and Technology, Department of Chemistry, Faculty of Sciences, Aristotle University of Thessaloniki, GR-54214 Thessaloniki, Macedonia, Greece; evouvoud@chem.auth.gr

**Keywords:** polydimethacrylate resins, dental restorations, dental decay, Bis-GMA, photopolymerization, nanomaterials, polymerization shrinkage, flowability, radiopacity

## Abstract

A review article has been conducted including the main research results and comments referring to flowable dental polymeric materials. To begin with, the synthesis and composition of this category of composites is discussed, revealing the major components of the commercial products in terms of chemistry and proportion. Later, the polymerization characteristics are unfolded regarding the reaction time and rate, volumetric shrinkage and depth of cure for both photocurable and self-curable composites. To continue, some perspectives of the pre-treatment or accompanying processes that a clinician may follow to enhance the materials’ performance are described. Fluidity is certainly associated with the progress of polymerization and the in-depth conversion of monomers to a polymeric network. Last, the aspects of radiopacity and translucency are commented on, showing that all flowable polymeric composites satisfy the radiography rule, while the masking ability depends on the fillers’ properties and specimen thickness. The reviewing article is addressed to all field scientists and practitioners dealing with flowable dental composites studies or applications.

## 1. Introduction

As often stated, the immense strides of the materials and auxiliaries used in dentistry in recent decades are due to the evolution of the chemistry and technology of polymers [1]. There are several categories of applications in dentistry where polymers and polymeric composite materials may find their place, such as prosthetics, restorations, surgical aids, impression surfaces, orthodontic accessories or periodontal cures [1]. Considerable academic research and clinical studies have been conducted on the most frequent type of interventions, the restorative polymeric materials. Since 1962 and Bowen’s resin (Bis-GMA), tremendous progress has taken place in terms of resins’ chemistry, fillers’ particle size and special ingredients or the curing devices applied, in order to achieve reliable restorations. With a view to enhance the final result, polymeric restorative materials today include, apart from conventional resin composites, glass ionomer cements, adhesives/liners, pre-treatment agents and flowable materials. A misconception is often observed regarding the terms describing subcategories of restorative composite materials, probably justified because of the continuous release of new products on the part of dental companies, not always with distinguished characteristics or targeted use. “Conventional” or “traditional resin-based composites” are placed in successive layers no greater than 2 mm in thickness. This time-consuming technique demands the clinician’s expertise; thus, the “bulk-fill” and the “packable resin-based composites” were developed, which can fill up to 5 mm at once satisfactorily [2]. “Universal dental composites”, as the word implies, offer a wider range of applications, an older tactic in the commerce of dental products. “Packable composites” are materials of high stiffness that are developed to behave more like amalgams, bearing higher filler content than conventional composites [3]. The viscosity modification of resin composites to the opposite direction led to “flowable resin-based composites”, with the augmentation of resin content [2]. Flowable resin composites chronically precede the bulk-fill composites. Careful use of the terms is required to keep the basic terminology accurate and targeted.

More particularly, “flowable resin-based composites” are restorative polymeric materials of low viscosity, through lowering the filler load (37–53%vol.) and/or by intergrading less viscous monomers. They are referred to as “cavity liners” or “dental primers” as well, since another main restorative composite follows them. The first generation of flowable bulk-fill composites were designed to be placed in a single increment followed by the covering with the conventional restoratives, which were also found then as “capping materials” [3]. Many studies today declare that the second generation of flowable composites do not differ from resin restorative composites in terms of clinical performance; thus, flowable materials may be used as complete restoratives (class II) in certain cavity cases [3,4,5]. The primary function of flowable composites remains to bond the subsequent polymeric restoration on the enamel, effectively providing durable results [6].

Most manufacturers package flowable composites in small syringes with thin and small curved nozzles that facilitate dispensing in cavities and spots of various shapes [7]. The majority of dental composites are photopolymerizable, and some are self-polymerizable (two pastes are mixed under ambient conditions and soon form the hardened composite). Many commercial self-adhesive resin-based materials commercialized to date are flowable [8]. The advantage of self-polymerizable composites is that they allow progression in curing into a cavity, ensuring retention even onto spots that light cannot penetrate [3]. In general, apart from their conjoining role, other applications that require material in injectable form may be shown, and these are summarized in Table 1 [7].

The aspects which determine the good performance of the flowable resin composites may be listed as: the filler percentage and the viscosity; the composition of the monomers and other ingredients; the polymerization shrinkage and marginal adaptation; the method of the hand application; the device applied and the clinician’s expertise; the proper cleaning, etching and drying of the cavity; the thermomechanical tolerance and physiochemical parameters of the materials; the colour stability and overall wear resistance; the former condition of the surface and the caries existing; and the patient’s oral hygiene and life habits [9,13]. The ingredients’ response towards those parameters builds the character of each product and finalizes its performance; thus, they are worth being reviewed and evaluated. However, it is useful to note in advance that research on dental materials is divided into two categories concerning the origin of the materials: the experimental syntheses and the commercially acquired products. This is crucial regarding the greater range of measurements and applications within which commercialized products are investigated, compared to the experimental products, which are more restrained.

The overall advantages of using flowable polymeric dental materials appear to be: their high viscosity that facilitates adjustment to all irregularities, the avoidance of air bubbles due to minimum thickness and flow, aesthetic restorations and colour match, radio-translucency for future radiography needs, and biocompatibility and acceptable resistance. On the other hand, the mechanical behaviour, polymerization shrinkage, water sorption characteristics and life-expectancy stand out as the main drawbacks of flowables [7].

The aim of this review article is to summarize and briefly present the progress that has taken place over the last decade (2012–2022) in the field of dental flowable polymeric composites, in terms of composition, properties, stability and resistance. Since most of the literature places an emphasis on restorative materials (class II), less research work has been carried out for flowable materials and few recapitulation efforts exist in the literature. Thus, a comprehensive evaluation of the scientific findings proves to be beneficial in helping to identify the factors affecting the final performance of the flowable dental composites.

## 2. Materials and Methods

An electronic literature search was conducted until June 2022 on the Science Direct, Scopus and Google Scholar search engines. The search terms engaged were “dental flowable polymeric composite materials” and the engines used the OR to connect those words. The articles appeared in order of relevance, by locating those words (especially the word “flowable”) in the title of the publication as preferable, then the keywords/abstract of the document. The time range of the publications gathered was limited to between 2012 and 2022, i.e., this publication revises the recent research on the topic. Duplicates of a paper found in different webpages were removed. All articles published in the English language were eligible to be included in this review. Unpublished articles in press, pending patents, personal communications, manufacturer advertisements, etc., were excluded (filtered out). The limitation of the present study is that the production of publications that could be included in this review is constant, thus, searching had to end at some point in order to proceed with the text.

**Figure 1:**
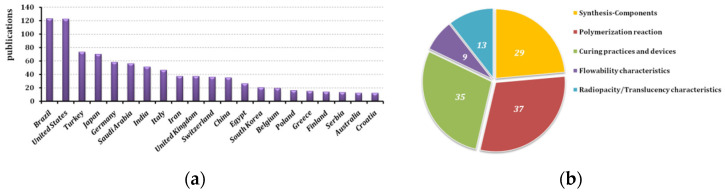
(**a**) Distribution of relative literature based on the country of origin for 2012–2022 period (scopus.com); (**b**) distribution (by the author) of the papers found on the topics discussed in the present publication.

Finally, 776 relevant articles were retrieved, and the statistics concerning their characteristics were recorded. The articles belonged to 160 scientific journals and covered 65 countries of origin in total, the primary ones being shown in Figure 1a. Then, the author conducted a final screening of the articles that were to be reviewed, by briefly reading the documents in order to evaluate their relevance to the publication to be written. A total of 105 articles were chosen and distributed in five subsections that will be presented and discussed. The distribution of the articles in those categories is shown in Figure 1b (naturally, there were overlaps in some cases).

## 3. Development of Discussion

This section is divided into five paragraphs commenting on defining aspects of the issue, such as the nature of the flowable composite to be used, the process to be followed for its application and the curing settings. Alongside this, information is given on flowability itself, what makes these composites different, and the radiopacity that is required.

### 3.1. Synthesis and Components of Flowable Materials

Regarding all dental restorative composites, dimethacrylate monomers build the resinous matrix and silicate fillers of various particle sizes form the inorganic phase. Additionally, the initiation system, pigments for shade, coupling agent and stabilizers are also essential ingredients in less than 1%wt [1].

Starting with the monomers included, Bis-GMA (rigid monomer) and TEGDMA, UDMA (diluents monomer) remain the commoner dimethacrylates that are applied (35–45%wt in total) and found on the market. An interesting alternative is proposed by Catel et al., synthesizing acidic vinylcyclopropanes, which follow a ring-opening reaction through radical mechanism. Then, polymerization may occur through radical reactions and crosslinking through the reaction on the unsaturated bond that is formed. Certainly, bis(4-methoxybenzoyl)diethylgermanium is proposed as the proper photo-initiator in that case along with CQ/tertiary amine. Flowable composites containing these acidic vinylcyclopropanes proved promising alternatives, since the replacement of methacrylates by vinylcyclopropanes led to a significant reduction in the shrinkage stress for their case [14]. Other, less known acrylates may exist in commercial products, such as Filtek^TM^ Supreme Ultra Flowable Restorative (3M ESPE, St. Paul, MN, USA), which is enhanced by 2,2-bis [4-(3-methacryloxypropoxy)phenyl]propane in its resin system [15]. Another example is SureFil SDR flow (Dentsply, DeTrey GmbH) containing a novel UDMA-based monomer with high MW, which helps to reduce shrinkage. The novel part of the monomer consists of photoactive groups embedded in the backbone of oligomeric species. The rationale is that under light the photoactive groups undergo photo-cleavage and generate radicals, which can further contribute to the overall crosslinking of the material. Indeed, these materials have been shown to reduce polymerization stress without reducing the R_p_% or DC%. Theoretically, this would eliminate the need for incremental filling on the basis of stress reduction [8]. Furthermore, siloranes are hybrid systems that contain both siloxane (known for their hydrophilicity) and oxirane-based monomers (known for their low shrinkage and stability). Maia et al., in their study concluded that the flowable silorane-based resin exhibited the least polymerization shrinkage among other flowable polymeric composites. Although siloranes are not often on the market in terms of matrix, a low shrinkage flowable composite performed similarly to a non-flowable one [16]. Among various novel monomers found in commercial products, glycero-phosphate dimethacrylate (GPDM) is also common.

Regarding the traditional initiation system for dental acrylates, a/tertiary amine, it is concluded that the photo-initiator concentration affects the DC%, mechanical properties and colour parameters of flowable composites. Composite containing 0.25%wt CQ showed lower elastic modulus E’ and shrinkage stress when compared to other concentrations (0.50, 1, 1.50 and 2%wt). Depth of cure (DoC) was 3 mm for composite containing 1%wt CQ. In addition, the increase in the concentration of CQ is directly associated with the augmentation in the DC% and E΄ values of flowable composites [17]. Boboia et al. in their research studied two mechanisms of initiation: self-curing through *N*,*N*-dihydroxyethyl-*p*-toluidine/BPO, and photocuring through CQ/DMAEM. It was found that the DC% achieved plays a key role in the water sorption and solubility parameters, while the mechanical correspondence depends on the filler characteristics [18]. Peterson et al., concluded that self-adhesive flowable composites present low bond strengths on enamel and dentin and, therefore, cannot be recommended for direct filling material, but in combination with restorative nanocomposites [19]. Tuloglou et al., agree on their case, in a clinical study, since it was found that the self-adhering flowable composite illustrated lower bond strength values than conventional flowable resin composite for both primary and permanent dentin treatments [20].

The inorganic phase remains lower in flowable materials compared to the packable restorative composites, in favour of fluidity, accepting the poorer mechanical response. SiO_2_, Sr-, Ba-, Na-, Al-silicates, fluorinated glass, prepolymerized resins, ceramics, TiO_2_, various metal oxides and others are found among the fillers used in dental composites [18]. The higher the inorganic phase rises (55–65%wt), the greater the mechanical characteristics appear. The differences in the strength of different types of dental flowable composites are due to their size distribution. The very small particles inserted contribute to the reduction in the fracture incidence. The appropriate distribution (i.e., hybrid composites) of fillers produces higher mechanical properties [18]. Dafar et al., incorporated nanotubes of TiO_2_ (0.5, 1, 1.5, 2, 3, 5%wt) in a commercial flowable dental composite by hand-spatulation on a glass plate and found that the dynamic Young’s modulus and fracture toughness were improved with a minimum decrease in the flowability and radiopacity of the composite. The functionalization of *n*-TiO_2_ with methacrylic acid is crucial for being attached chemically with the polymeric matrix [15]. Other results indicate that commercial flowable composite mixed with laminate ceramics (SiO_2_, BaO, Al_2_O_3_, CaO, CeO_2_, Na_2_O, K_2_O, B_2_O_3_, MgO, ZrO_2_, P_2_O_5_, TiO_2_ and pigments) works fine in a minimum-only thickness, without great impact on the DC, yet without reaching a DC similar to the control group [21]. Raju et al., prepared their own flowable composites with the aim of identifying the influence of the glass fibres in them. The usage of short glass fibres in flowable bulk-filled composites has enabled a reduction in incremental steps and increased ease of placement, while maintaining mechanical properties. With the replacement of 5 wt% fillers with a smaller size ratio, DoC increased due to higher DC% because of the enhanced light penetration in the material. However, fibre replacement had a detrimental effect on the volumetric shrinkage and a negligible effect on water sorption and solubility [22].

The coupling agent (the silanes) consolidates chemically, through covalent bonds, the polymeric matrix and the filler particles, which are covered by it in monolayers. The common *γ*-MPS (or MPTMS) may be used, as is, in experimental or commercially available products. It was found that there were differences in the reactivity of the silanes with polished and acid-etched ceramic surfaces. Hydrolysis reactions are important in the formation of Si−O−Si bridges with inorganic particles. The silane primers and adhesives contain various forms of MPTMS, including partially hydrolyzed silanol monomers, methoxy- and methoxysilanol-functionalized siloxane dimers and siloxane polymers. They affect the bonding capability among the flowable materials, as well as the restorative composites. The chemical bonding capacity of the silanes is highest in products with silanol monomers. Acid-etching (5%vol HF) increases bond strength to a level that balances out the reduced chemical bonding capacity of several products with reduced or minimal silanol activity [23]. Another interesting recent paper explores the influence of an optimum amount of silane to improve the interfacial adhesion at the fibre–matrix interfaces, where 3-(glycidyloxy)propyltrimethoxy silane (3-GPS) and 8-(methacryloxy)octyltrimethoxy silane (8-MOTS) were used apart from traditional MPTMS. The study showed that the hydrophilicity properties of the fibres are affected by the concentration of the silane coating the fibres. Moreover, the optimum molar %/wt concentration of silane improves the adhesion between glass fibre and epoxy matrix. Resin composites reinforced with optimal concentrations of 8-MOTS-grafted glass fibres showed a slight increase in mechanical properties [24].

A Zn-doped phosphate-based glass was applied to dope a flowable resin and evaluated the antibacterial activity of the composite against *Streptococcus mutans* (*S. mutans*) to extrapolate the preventative effect toward secondary caries, where the antibacterial activity was enhanced [25].

The researchers and field practitioners should always keep in mind that the overall material behaviour and characteristic properties are an outcome of the composition, i.e., the ingredients included and the proportions. Even slight changes in their composition may alter their character.

### 3.2. Polymerization Reaction and Characteristics

Radical polymerization is the route for the curing of dimethacrylates, mostly through the mechanism of free radicals. The techniques applied are either photopolymerization or self-polymerization, depending on the initiation system that the composite bears. The cross-linking (“curing” or “hardening”) through the second vinyl bond of the monomers occurs soon after the addition of the molecules to the macrochains, which is why DC is proper instead of a degree of polymerization (DP%), terms that are not identical for those polymers.

Most scientists follow the manufacturers’ guidelines regarding the photopolymerization time, but investigations show that this is not something granted. Manufacturers’ recommendations concerning curing directions could result in a lower final setting. In most cases, if curing time is longer than the one indicated by the manufacturers, higher characteristics will be achieved. Subsequently, manufacturers’ recommendation regarding the curing time should be re-evaluated and updated based on the results of the current literature/research (pe. 20 s extension with a 1000 mW/cm^2^ curing unit or doubling the proposed time) [26,27,28]. Although some of the tested materials exhibit good performance, it is still recommended to “cap” the flowable bulk-fill materials with a 2-mm layer of conventional composite to prevent the subsequent solubility of the composite material [26]. Anad Yokesh in his research studied two commercial flowable composites and found that the one consisting of urethane-based monomers presents higher DC and DoC values, compared to another bearing a stiffer structure in the matrix, because of Bis-GMA and Bis-EMA monomers [29]. As Arenas Buelvas et al., comments, the material’s viscosity interferes with its porosity too, as well as in response to the irradiation process (pe. with 1000 mW/cm^2^ for 20 s or with 3200 mW/cm^2^ for 6 s). Furthermore, in many cases, the greater the polymerization shrinkage strain, the lower the porosity. On the other hand, the synergistic combination of different photo-initiators allows the polymerization process of the methacrylic monomers to occur more efficiently, increasing the final DC [30].

Τhe presence of light-absorbing photo-initiators in resin composites results in the attenuation of the light intensity along the radiation path and often limits the DoC of these materials (partly, since most of the light attenuation occurs due to intense light scattering on filler particles). In other words, a high CQ/amine concentration may result in the generation of a very high concentration of free radicals, of which only a fraction may participate in the polymerization reaction [17]. The optimal concentration of CQ in Bis-GMA/TEGDMA flowable composite is ≤1%wt of the resin matrix, since this CQ concentration allows adequate balance among the DC, DoC, mechanical properties and colour characteristics of these materials [17].

A samples’ bottom/top scraping-away study conducted by Garcia et al., stated that, for top hardness measurements, all the materials exhibited similar values, regardless of sample thickness, whereas for bottom hardness measurements, all the materials showed a decrease in hardness with increasing depth. Nevertheless, the numbers depend highly on the composition and percentage of the matrix in the flowable composite [31]. Similarly, the increase in the irradiation time results in an increase in the micro-hardness, mainly on the bottom surface of the flowable composites. It is noteworthy that under the same polymerization conditions (light intensity and irradiation time, composite thickness and colour, LCU tip distance and angulation), different values are obtained for the different composites, among which the flowable achieve the lowest micro-hardness values [32]. On the other hand, while the composites with lower filler content (low viscosity) are generally considered to have higher polymerization shrinkage than the conventional packable composites, a study has shown that polymerization shrinkage is dependent upon the compliance of the testing instrument. In detail, the desired compliance of the testing instrument was achieved by varying the sample position along the cantilever beam. The high compliance of the constraint eliminated the difference in the shrinkage between the bulk-fill flowable and packable composites. Nevertheless, the bulk-fill flowable composite developed less shrinkage percentages than the packable composite under lower compliances [33]. Bulk-fill flowables are properly cured in 4-mm bulk, but they shrink more than the conventional non-flowable composites [34]. The measurement of polymerization shrinkage stress depends on the compliance of the testing device. Ku et al., investigated 12 flowable composites from six manufacturers, divided into groups of high or low flowability, according to the manufacturers’ claim. Those of high flowability showed significantly higher polymerization shrinkage, regardless of the light-curing unit used [35]. In another study, the incremental filling with a flowable composite liner showed higher spot deflection than that without a flowable liner, and the difference was maintained until 2000 s after the initiation of light curing. This phenomenon could be explained by the high shrinkage strain of flowable composite, which is a major factor in producing stress in a high compliance situation. It is suggested that teeth with prepared cavities have some compliance, and that the testing system should have compliance that is comparable to that of a tooth, in order to perform clinically relevant studies [36]. For instance, compared to the Z350 flowable composite with a filler content of 65 wt%, Z250 conventional composite (both Filtek™ Supreme, 3M ESPE, USA) with a filler content of 82 wt% showed a significantly lower shrinkage strain of 0.70% [37]. The low shrinkage flowable composite performed similarly to non-flowable composite with a significant difference compared to the other flowable resin-based composites, according to Maia et al. [16].

The extent of post-irradiation polymerization in dental composites depends on the reaction that takes place during light exposure. In the case of a rapid reaction, parts of the conversion process end immediately after light exposure, leading to reduced post-irradiation polymerization, when the layer is thin, and the light-emission is strong. On the other hand, in the case of a medium advance of the reaction, increased post-irradiation polymerization is noticed for most matrixes. Hirata et al., in their research noticed that the use of dental adhesive decreased the overall volumetric shrinkage of flowable composite. They speculated that since these materials present higher viscoelasticity compared to conventional composites, they may better dissipate polymerization shrinkage stress when bonded to the cavity walls, reducing the gaps around the restoration surfaces. It is claimed that for a strongly attached composite restoration, the shrinkage vectors are oriented down and toward the bonded margins, which may explain the misfit of the flowable composite studied and its volume reduced at the occlusal surface observed in the 3D reconstruction Another explanation of the influence of the adhesive in flowable material is the delay of the gel-point (cross-linking), resulting in a slow polymerization rate of the flowable composite compared to a packable paste, thereby allowing deformation without overstressing areas near the cavity walls (an explanation supported by the research findings) [38]. Post-curing is of crucial importance regarding the final monomers’ elution. The amount of released Bis-GMA and TEGDMA monomers (i.e., non-polymerized monomers) from the bulk-fill flowable composite materials was generally lower than from the conventional flowable composite [27,39]. Another study evaluated the post-curing shrinkage of flowable dental composites at DoCs of 0, 2.5 and 5 mm, and highlighted that the maximum shrinkage strain happens at the depth of 0 mm and the lowest shrinkage strain happens at the depth of 5 mm. A step further, it claims that DC% and rheological flow behaviour could be regarded as prognostic factors for understanding the post-polymerisation shrinkage strain trend in dental composites [40].

It is important to add that the DC% of photo-curable resin composites depends on the differences of refractive indexes, kinds of monomers or fillers, shape and distribution of fillers and photo polymerization initiators, etc., besides filler/matrix content [1]. A cavity base material is applied on the cavity wall and floor, and thus, the light guide tip cannot be placed close to the restored resin composite. Light intensity decreased with increasing the distance between the light guide tip and the surface of the resin composites. The decreased light intensity can be compensated for by extending the irradiation time. In the clinical situation, it is important to use a longer irradiation time for the polymerization of cavity base materials [41]. Another aspect was examined when Zn-doped glass was incorporated in flowable composite for enhancing its antimicrobial character; no significant difference in the DoC between groups was noticed, meaning that the incorporation of Zn-doped glass did not affect the polymerization of the composite [25]. Pereira et al. meticulously investigated the effect of curing on the traditionally used monomers in resin composite manufacturing, for which monomers proper DoCs were confirmed. It is claimed that the resin matrix interferes in the rheological behaviour, the translucency parameter and polymerization capacity as a function of depth. The Bis-EMA/UDMA-based materials demonstrated the highest curing potential as a function of DoC. The materials tested showed pseudoplastic and thixotropic behaviour and a predominance of viscous effects over elastic correspondence. First the Bis-EMA-based matrix, then Bis-EMA/UDMA-based, and finally, the UDMA-based group had the highest viscosity [42].

As known from theory, the polymerization rate, polymerization shrinkage, DC and DoC values are heavily influenced by the matrix ratio and synthesis in flowable dental composites. Then, the efficient curing is affected by the polymerization time, the potential of the device, any escorting practices and the addition of other adhesives. These parameters are examined in the next section, which aims to ameliorate the final result, on the clinician’s side.

### 3.3. Clinical Practices and Devices

What is the practice for the accurate marginal adaptation of flowable composite during restoration? What etching preparation is required for strong adhesion? What is the proper light-curing unit and does any external source of energy help the rise of DC? These are some of the issues that practitioners need to manage during their professional life for successful dental restorations.

Acid-etching (~30% H_3_PO_4_) is the commoner practice for local cavity pre-treatment, which aims to remove the smear layer and create roughness of the surface microscopically, increasing retention with sealant. It is worth noting that air-abrasion is a pseudo-mechanical method to increase roughness and achieve better retention [9].

Certainly, samples cured by LED were significantly stronger compared to that of QTH. The light-cure time mainly affects the hardening by the LED curing system. In the case of dental composite bonded with an adhesive resin, it can be enhanced with QTH light cure [43]. The greater the distance of the tip of the device from the surface, the lower the DC that is achieved [40]. Finan et al. tested the efficiency of irradiation in distances 1–8 mm from the composite surface and found that the bulk-fill flowable composite had a satisfactory DoC of 4 mm, confirmed by DC and hardness analyses [44].

Impressively, the real-time observation of composite placement and the 3D-quantification of interfacial gaps has been engaged. Interfacial gap formation during composite polymerization depended on the adhesive system used. The formed gaps continued to propagate after composite light-curing finished [45]. Another new instrument was able to measure the true linear shrinkage of composites without sensitivity to the specimen geometry and the viscosity of the material. Therefore, this instrument could be used to characterize the shrinkage kinetics for a wide range of commercial and experimental materials that are polymerizable with visible light, in relation to their composition and chemistry. This study developed a particle tracking system to measure the linear polymerization shrinkage of light-cured composites without direct contact with a specimen using a computer [37].

The incremental filling technique, which is the most common yields significantly lower cuspal deflection than the bulk-filling technique in the case of Filtek™ Supreme composites. Flowable composite lining under universal composite (Z250) layering showed higher cuspal deflection than that with no flowable composite lining [36]. A recent systematic publication of Ferracane et al., illustrated the placing methods for restorative materials (class II), including multiple thin increments, conventional filling, incremental fill with flowable liner, flowable bulk-fill liner composite with conventional composite capping layer or bulk-fill restorative, including sonic energy application and dual-cure (i.e., another technique that follows the photopolymerization to enforce the material’s performance). The outcome presented in the literature for these various approaches suggests that the most important factor for achieving success is careful and proper placement and the light-curing technique, independent of the placement approach [2,3]. Among the variables that Tabassum et al., studied, increment thickness has the greatest effect on DoC, while changes in device voltage have a minimal bearing on the DoC of flowable composite [46].

The literature also mentions that the Y-Al-garnet laser is one of the most useful types of lasers for dental hard tissues; it emits a wavelength that effectively treats the dentin surface, similarly to acid-etching. Cavities with a high surface quality are sharp and well defined, whereas the results show no significant differences regarding the microhardness of the self-etching composites and the laser-etching samples [47]. Another potential treatment is achieved through ultrasonics. The DC% increased with ultrasonic application for flowable composites, but only with 30 s ultrasonic activation. The voids were reduced linearly with ultrasonic application in flowable composites. The frequency and time of the ultrasonic application is an important factor to consider and can be engaged to preheat composites before clinical application [5,48]. The irradiance of the LCU seems to be a more important factor than heat development in the pulp chamber. Flowable bulk-fill composites can act as a pulpal insulator against LCU irradiation. Clinicians should consider the risk of pulp tissue overheating. LED LCU might generate a high temperature that could lead to malicious effects on the pulp tissue, especially when small increments of resin-based composites are used for restoration [49]. Often, no benefit is gained by heating the intervention after the initial light-curing. To avoid misconceptions, heating in the pulp area is not common, and it has been examined as an auxiliary mean. A heat treatment for larger sized photocured samples might still be valid (e.g., inlays, onlays, indirect restorations), whereas small restorations may well directly intraorally photopolymerize, without the need for a post-cure heat treatment. The flowable composite achieves a T_g_ that is as high as the hybrid composites or nanocomposites demonstrate, indicating good photocuring at a certain thickness; however, its inferior thermal stability (as evidenced by a higher mass loss) precludes it from being subjected to much heat treatment after the initial photocure. It would, therefore, be advisable not to employ the flowable composite as part of an inlay/onlay restoration (associated with further heat supply on the material). It is suggested that the flowable material is best reserved for direct intraoral photocure only in a thickness of 2 mm maximum (as is the general rule for all composites) [50].

Despite the photopolymerization, dually curable composite materials remain necessary for thicker restorations. A high-irradiance LCU only has a limited effect on the maximum thickness of a CAM/CAD (computer aided designed/manufactured) material through which one can light cure [51].

Other types of auxiliaries are opaquer materials, for instance, those composed of inorganic filler particles such as zirconium silicate, various dimethacrylates (such as UDMA), stabilizer, pigments and initiators. Lümkemann et al., used an opaquer on an aryl-ketone polymer, i.e., coated with a thin layer of opaquer (Universal Opaque A30, SHOFU Dental Corp) that was applied with a brush in terms of mechanical endurance [52]. Typically, resin-based dental materials are required to be flowable or mouldable before setting and provide adequate mechanical strength after setting. The setting method may include light-curing, self-curing or heating. Dental adhesive bonds dental filling composites onto tooth tissue and has a very low viscosity, which helps it to be coated. Compared to other resin-based dental materials used in direct restorations, resin luting cement used in indirect restorations has the advantages of various curing methods. With the development of technology and the improvement of materials, the resin-based materials are continually being optimized and functionalized, playing an all-in-one role [53]. Finally, the resin-coating technique is one of the successful bonding techniques used for the indirect restorations. The dentin surfaces exposed after cavity preparation are coated with a thin film of a resin material combined with a flowable composite. Resin coating can minimize pulp irritation and improve the bond strength between a resinous cement and tooth structures, or even prevent root caries in elderly patients. Therefore, the coating materials have the potential to reinforce and preserve dental interventions [54].

Specialists are able to correlate this knowledge during material selection, manipulation and placement for the increased longevity of restorations.

### 3.4. Flowability Properties

The flow distance (cm) and flow rate (mm/s) of flowable composites are investigated: among the specimens, products of high flowability and low viscosity (filler content less than 60 wt%) showed greater values of flow distance and flow rate compared to those of low flowability and high viscosity products. Flowable dental polymeric composites are non-Newtonian, shear-thinning materials [7]. In this study, the filler contents showed a negligibly low correlation with the flow distance of the tested products, indicating the possibility of high dependence on the amount of each monomer (Bis-EMA, Bis-GMA, TEGDMA and UDMA) and their combinations [35]. Shouha et al., visually evaluated the viscosity of dental composite materials and characterized them as “low flow” (resin content 54% vol) that is transformed to “flowable” with 5%vol glass fibres incorporation, “paste” when glass fibres are added at 20–40%vol fraction and “packable” when the presence of the reinforcing agent reaches 60%vol [11]. The dispensing of fibrous inorganic phase in the flowable material influences the fluidity of the material, maybe less than in the case of particles.

The rheological properties of resin-based composites are related to filler volume, filler particle diameter and handling temperature. Compared to the general composites, the flowable composite filler has a lower load, better flowability and lower viscosity. The flowable composite has the advantages of easy handling and good permeability to the treating surface. Great teeth adhesion is achieved, its shrinkage rate is less than that of the common composite material and the curing depth is more satisfactory. Hence, the viscosity and flow characteristics of flowable resin composites can have a potential influence on their handling properties and, thus, on their clinical indications. As a result of differences in viscosity, flowable composites vary considerably in polymerization shrinkage, stiffness and other physical properties. Given the picture of viscosity, Bis-GMA presents *η* = 369 Pa·s, Bis-EMA_(30)_ *η* = 0.82 Pa·s, UDMA *η* = 7.04 Pa·s and for TEGDMA *η* = 0.008 Pa·s (in a temperature range of 20–25 °C) [53]. The Bis-GMA monomer has the lowest rheology, the addition of TEGDMA allows the reduction in the viscosity, while the addition of Bis-EMA and UDMA increased the overall viscosity [42].

Small molecule monomers such as TEGDMA that lack H-bond proton donors present very low viscosities, unlike Bis-GMA [30]. Amounts of TEGDMA are leachable from flowable composites, since residual uncured monomers or oligomers are evident in the matrix. Resin-based materials continue to release measurable amounts of composite components beyond the initial 24-h period, although the rate of release decreases with time [55].

Finally, as far as the various adhesives are concerned, the low-viscosity liners could provide better wettability/adaptation on the depth of cavities. Yet, it was demonstrated that a flowable liner probably bonds and polymerizes simultaneously, leading to viscoelastic flow that reduces the negative effect of polymerization stress on the bonding. This property is advantageous in terms of marginal adaptation [56].

### 3.5. Radiopacity and Translucency Characteristics

Radiopacity facilitates diagnostic observations that are adjacent to flowable resin composites. The radiopacity of dental restorative materials is a result of the type, percentage and proportional amount of the radiopaque element (Zn, Zr, Sr, Ba, La) in the filler part, rather than radiolucent (silica, silicates). A lower filler content is the reason for lower radiopacity compared to hybrid resin composites. The desirable radiopacity of resin composites is still a controversial issue. Materials with radiopacity lower than enamel might be misinterpreted as secondary caries on radiographic images [7]. When the radiopacity is too high it may obscure details of adjacent anatomy, and excessive radiopacity may hide the diagnosis of caries adjacent to the restoration [57]. Radiopacity is often declared by manufacturers for each flowable composite material. Researchers should know that the radiopacity of dentin is 1.09 ± 0.0, and enamel 1.84 ± 0.0 mm eq. Al [58]. A study applying digital radiography proved that for all investigated bulk-fill flowable composites, significantly higher radiopacity values were evident in comparison with those of enamel, dentin and most of the conventional flowable composites (among them a self-curing material too). The majority of the manufactures’ values were close to the results of the studies; thus, it is concluded that the radiopacity data provided by the manufacturers can be trusted [59]. The incorporation of 3% *n*-TiO_2_ in flowable composite slightly diminished the radiopacity values. Moreover, the non-functionalized samples behaved better than the functionalized ones [15].

The digital radiopacity analysis techniques used in research provide an easy, reliable, rapid and precise method to characterize the radiopacity of dental flowable composite materials. Dukic et al., investigated about 20 commercial flowable composites and all of the tested materials had higher radiopacities than dentin, but at almost every combination of exposure and voltage, there were also some materials with radiopacities equal to or slightly greater than enamel [60]. The Tetric^®^ family of products (Ivoclar Vivadent, Schaan, Liechtenstein), for instance, contain Y- or Yb-compounds (*pe*. YbF_3_), which are responsible for great radiopacity.

Regarding the radiopacity of CAM/CAD materials, a careful approach should be explored. At a 1-mm thickness, the radiopacities of CAD/CAM specimens matched or were slightly lower than enamel. At a 2-mm thickness, the resin composite blocks were significantly more radiopaque than the ceramics. The radiopacity of polymer-infiltrated ceramics was low, despite the presence of radio-pacifying elements and high filler. This study, also providing great images, confirmed the joint influence of composition and thickness on radiopacity. CAD/CAM restorative materials showed thickness-dependent radiopacity development [61].

Bleaching shade composites have been introduced to facilitate matching with shades resulting from tooth bleaching procedures. Since the bleaching shades of nano-flowable composites are relatively new, translucency parameters are useful to be calculated (by spectral results). The polymerization itself may change the transparency of a flowable composite layer, since ketone/quinone groups may be activated, towards darker/duller shades. Because of the higher opacity, the DC% of white shades is lower than darker shades, which may also result in a greater colour/shade shift. Thus, for some flowable composites that are more vulnerable to opacity changes than conventional composites, the ageing/wear process results in slight increases in the translucency parameter [62]. Another aspect is that for the low-viscosity resin-based composites, when increasing the pulpal temperature, the more translucent ones showed a significantly higher temperature change [28]. Translucency is determined not only by the great parts, such as matrix, filler composition and content (in general, the higher the crystallinity the lower the translucency [12]), but also by relatively minor pigment additions and potentially by all the chemical components of these materials.

Flowable bulk-filling composites (which have been launched for usage in thick layers) have different masking abilities than conventional universal filling materials, which clinicians should take into account in order to achieve optimum colour matching and aesthetic results (the layering technique is proposed) [63]. For the case of luting agents and veneers, the more translucent and light-shaded they are, the more perceptible is the shade change of the luting agent [12]. Finally, the effects of thickness on the light transmittance, opalescence and translucency in the dental composite restorations are given. For instance, the average transmittance, opalescence and translucency parameters were marginally influenced for specimens between 0.2 and 0.6 mm thick [64].

## 4. Research Tools for Reliable Results

A brief note regarding the useful “tools” that a relevant researcher engages when dental polymeric materials is the case. When revising several studies or working on dental polymeric materials, some instructions, comparisons or statistical practices are followed often. ISO 4049/2019 (fifth edition) “specifies requirements for dental polymer-based restorative materials supplied in a form suitable for mechanical mixing, hand-mixing, or intra-oral and extra-oral external energy activation, and intended for use primarily for the direct or indirect restoration of the teeth and for luting” and consists of a necessary guide for experimental procedures and examination of the acceptable values in the results collected. ANOVA is a statistical process, as a (one- or two-) way to determine whether experiment results are significant compared to others. In other words, we decide whether we need to reject the null hypothesis or accept the alternate hypothesis. It is common in dental research for the authors to build hypotheses, correlating the experimental parameters, which may be verified or rejected. Tukey’s range test is a single-step multiple comparison that is used to find means that are significantly different from each other. The Kolmogorov–Smirnov test is a non-parametric statistical test of the equality of continuous one-dimensional probability distributions, which is used to compare a sample with a reference probability distribution or to compare two samples. The FDA (USA) is responsible for protecting public health; thus, it regulates the levels of drugs and all chemical compounds for humans, other biological products, medical devices, cosmetics, etc. Therefore, scientists ought to take into consideration the regulations and limitations that national/international committees pose for biomaterials.

## 5. Conclusions and Future Trends

This article summarizes major aspects found in the relative literature (2012–2022) concerning flowable polymeric composites for dental applications, and herein, five areas are included, presenting their preparation and handling. Particularly, the ingredients engaged in the synthesis of those materials and their composition are demonstrated; the initiation system and special ingredients incorporated, such as radiopacity elements, are also discussed. The changes in the degree of conversion and depth of cure are explained based on the composite used, the light unit applied, or the irradiation time executed on a given specimen. It was concluded that flowability is a determinant parameter for satisfactory curing or the monomers’ solubility over time.

As for the direction of current research, I would oppose the development of novel monomer structure and the synthesis of resins that would shrink less volumetrically after polymerization. Secondly, seeking the best combination of materials or techniques for the adequate marginal adaption is ongoing. Finally, the correlation between viscosity/fluidity and the degree of conversion remains hard to evaluate.

## Figures and Tables

**Table 1:** Summary of the dental uses where flowable polymeric material finds applications.

Application Category	Specific Uses
Restorative materials	in paediatric dentistry, as main restoratives [6] in preventive restorations (class I) as minimal class III restorations in abfraction cases (class V) as pit and fissure sealants [9]
Prosthetic materials	for denture repairs for bonding constructions of indirect prosthetics’ maintenance
Orthodontic materials	bonding brackets and retainers wire stoppers, molar stops [10]
Oral surgical materials	auxiliary material in endodontic treatments protecting patient’s mucosa against irritation [10] in emergency teeth reattachments [11]
Other applications	as luting agents and resin veneers [12] repair of small porcelain fractures for amalgam ditches or covering crown margins

## Abbreviations

Bis-GMA: (bisphenol A-glycidyl methacrylate); GIC: glass ionomer cement; LCU: light-curing unit; LED: laser-emitting diode device; ANOVA: analysis of variance; Bis-EMA: ethoxylated bisphenol A dimethacrylate; BPO: benzoyl peroxide; CQ: camphoroquinone; DC: degree of conversion; DoC: depth of cure; FDA: Federal Drug Administration; GPDM: glycero-phosphate dimethacrylate; MPTMS: (3-mercaptopropyl)trimethoxysilane; QTH: quartz-tungsten-halogen device; TEGDMA: triethyleneglycol-dimethacrylate; UDMA: urethane dimethacrylate.
